# Zika Virus (ZIKV): A New Perspective on the Nanomechanical and Structural Properties

**DOI:** 10.3390/v14081727

**Published:** 2022-08-05

**Authors:** Maria Luiza de Araujo Dorneles, Ruana Cardoso-Lima, Pedro Filho Noronha Souza, Daniela Santoro Rosa, Tais Monteiro Magne, Ralph Santos-Oliveira, Luciana Magalhães Rebelo Alencar

**Affiliations:** 1 Laboratory of Biophysics and Nanosystems, Physics Department, Federal University of Maranhão, São Luís 65020070, Brazil; 2 Department of Biochemistry, Federal University of Ceará, Fortaleza 60440900, Brazil; 3 Drug Research and Development Center, Department of Physiology and Pharmacology, Federal University of Ceará, Fortaleza 60440900, Brazil; 4 Department of Microbiology, Immunology, and Parasitology, Federal University of São Paulo, São Paulo 04023062, Brazil; 5 Brazilian Nuclear Energy Commission, Nuclear Engineering Institute, Rio de Janeiro 21941906, Brazil; 6 Laboratory of Nanoradiopharmacy, Rio de Janeiro State University, Rio de Janeiro 23070200, Brazil

**Keywords:** ZIKV, physical virology, ultrastructure, AFM, arbovirus

## Abstract

Zika virus (ZIKV) is an arthropod-borne virus (arbovirus) from Flavivirus. In 2015, Brazil and other Latin American countries experienced an outbreak of ZIKV infections associated with severe neurological disorders such as Guillain–Barre syndrome (GBS), encephalopathy, and encephalitis. Here, a complete mechanical and structural analysis of the ZIKV has been performed using Atomic Force Microscopy (AFM). AFM analysis corroborated the virus mean size (~50 nm) and icosahedral geometry and revealed high mechanical resistance of both: the viral surface particle (~200 kPa) and its internal content (~800 kPa). The analysis demonstrated the detailed organization of the nucleocapsid structure (such as RNA strips). An interesting finding was the discovery that ZIKV has no surface self-assembling property. These results can contribute to the development of future treatment candidates and circumscribe the magnitude of viral transmission.

## 1. Introduction

The Zika virus (ZIKV) is a member of the Flaviviridae family of the Flavivirus genus, which groups more than 70 viruses, whose structural arrangement is similar to the dengue, yellow fever, and chikungunya viruses [1,2,3]. Zika is an enveloped virus [4,5] of a protein nature, which in turn is covered by a membrane (M) [6], which protects the RNA that is enclosed in a capsid (C) formed by an icosahedral set of proteins [7]. It was first isolated in 1947 in the Zica forest in Uganda. However, in 2007, after an outbreak in the South Pacific islands, it was considered an emerging pathogen by the World Health Organization (WHO) [8]. Its circulation has already been dated in more than 70 countries [9], and Epidemics at a global level break out, representing an intangible threat to public health, given that the number of infected individuals only grows. The main transmission route of this virus occurs during the hematophagy process of the infected female *Aedes aegypti* and *Aedes albopictus* mosquitoes [10]. This fact demonstrates the great potential for dissemination and the ability of the virus to propagate on a large scale inherent to a vector that acts as a kind of biological weapon [11]. The virus, when taking possession of the host body—considering its structural composition (enveloped virus)—acts as a master key capable of mimicking, during the replication process, proteins that resemble host cells. This makes it difficult for the immune system to respond in the replication process, which facilitates the spread of viral load to organs, muscles, and brain [12,13]. In contrast, despite the symptomatic similarities between the Zika and Dengue viruses, recent reports have suggested that ZIKV can sustain itself under adverse conditions. The reported current transmission cases through sexual contact, blood transfusion, and even transplants have been reported [14].

ZIKV has a replication that involves the introduction of viral nucleic acids and viral proteins inside the crude endoplasmic reticulum (ER), which are attached for cell maturation and release [15]. This feature guarantees the virus its ability to infect neurological cells (neurotropic flaviviruses). The spread of the virus has been linked with several other types of diseases, such as Guillain–Barré syndrome [16], meningoencephalitis [17], myelitis, ophthalmological abnormalities [18], and the frightening microcephaly [17,19], which in infected pregnant women, causes serious neurological complications to the fetus [20]. This highlights the relevance of this path of research dealing with a scenario that is not prepared to deal with possible new mutations of the disease, as there are currently no specific antiviral treatments or vaccines [21]. The current gaps are related to divergences in the literature, requiring more robust methods to evaluate these causal relationships. In this scenario, characterization by Atomic Force Microscopy emerges as a tool capable of “invading” the dome where this small infectious agent is found. The (until now) intangible viral layers of the ZIKV were characterized in this work, exploring nanomechanical properties such as elasticity, brittleness, or fatigue points of this Flavivirus. The AFM provides information on the structure and biological interactions, adsorption properties, membrane structure, and resistance of ZIKV, bringing a new perspective on viral particles [22]. From this, it is expected to contribute to a better understanding of cellular infection, seeking more information to develop future treatment candidates and circumscribe the magnitude of viral transmission.

## 2. Methodology

### 2.1. Virus Culture and Inactivation

Zika virus (Brazilian isolate, ZIKV^BR^) [23] was cultured in Vero E6 cells (ATCC, C1008) and titrated in Vero cells (ATCC, CCL81) as described by Moser and coworkers [24]. The virus was inactivated by thermal treatment at 56 °C for 1 h.

### 2.2. Atomic Force Microscopy (AFM)

AFM measurements were performed according to the methodology employed by Cardoso-Lima et al. [22], where 10 µL of solution with viral particle suspensions were deposited on glass slides (13 mm diameter). The slides were previously treated with poly-L-lysine 1% (Sigma, St. Louis, MO, USA) in order to facilitate the adherence of ZIKV particles to surfaces of glass slides [25] (only for fluid measurements), by the phenomenon of interaction between the negatively charged viral particles and between the polymers present in the positively charged poly-l-lysine [26]. The slides were analyzed on Multimode 8 (Bruker, Santa Barbara, CA, USA) and the probes used were SNL (Bruker) with 0.06 N/m nominal spring constant with a 2 nm radius in the peak force quantitative nanomechanics (QNM) mode. The structural parameters of the viral particles were calculated using the Gwyddion 2.57 software, applying the boundary grain detection to the topographic images (2 μm^2^ scan area). From these regions, statistical information on the height and diameter of 107 particles was calculated. The diameter measurements were performed under ambient conditions with dried samples. The influence of these conditions was not considered for the diameter measured because all the measurements were performed under 4 h after the sample was prepared, following the protocol performed by Oropesa et al. [27] for the measurement of dried Virus-Like Particles using AFM. As for the influence of the tip radius, it was not considered because the radius is only about 3% of the size of the particle, which makes the tip radius influence negligible.

Viral particle indentation experiments were performed on nine different viral particles, and each one has undergone 30 to 70 indentation cycles. Adhesion maps were analyzed on six viral particles.

For the indentation analysis, the measurements were performed on the QNM Ramp Mode in fluid following the same procedure used by Cardoso-Lima et al. [22]. We applied a force setpoint of ~6 nN and a tip velocity of 100 nm/s. AFM data were analyzed, and the maps were obtained using Mountains SPIP8 and Nanoscope Analysis software.

## 3. Results and Discussion

**Figure 1:**
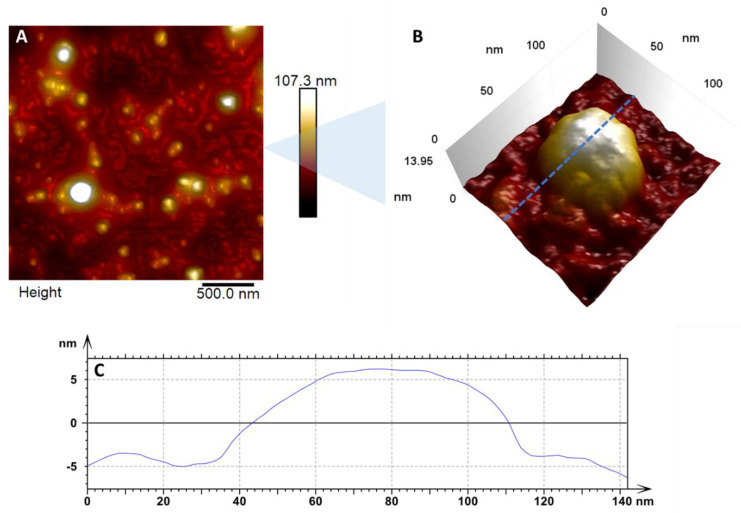
In (**A**) we have the enlarged topographic image of countless viral particles dispersed in the substrate, with a diameter varying between 47 nm and 69 nm. (**B**) Zoom of the surface of a single viral particle showing the details of the virus structure. The blue dashed line indicates the region where the cross-section shown in (**C**) was taken. (**C**) Cross-section of an adsorbed ZIKV particle in a glass substrate.

The first results come from the topographic maps (Figure 1), where it is possible to observe the details of the virus’s outer surface. In Figure 1A, a larger scan area exemplifies the density of particles in the sample studied. From a population of about 107 viral particles, we measured that each has a mean size of 54.53 nm with a standard deviation (SD) of 6.9 nm.

Although some reports have concluded that ZIKV has a size varying from 70 nm to 100 nm [28,29], our data demonstrated that the real size of the virus is 50 nm, as corroborated by Barreto-Vieira et al. [30], Sexton et al. [31], and Cui et al. [32]; the information must be updated. For the map in Figure 1B, we have a close-up look at the arrangement details on the virus’s surface. It is possible to observe small bumpy regions and well-organized proteins. This characteristic is expected for ZIKV [6]. In Figure 1C, we have a cross-section of a single viral particle, evidencing its height and profile. The diameter of the viral particle (width to half-height) is 65.4 nm and the height measured was 10.8 nm.

**Figure 2:**
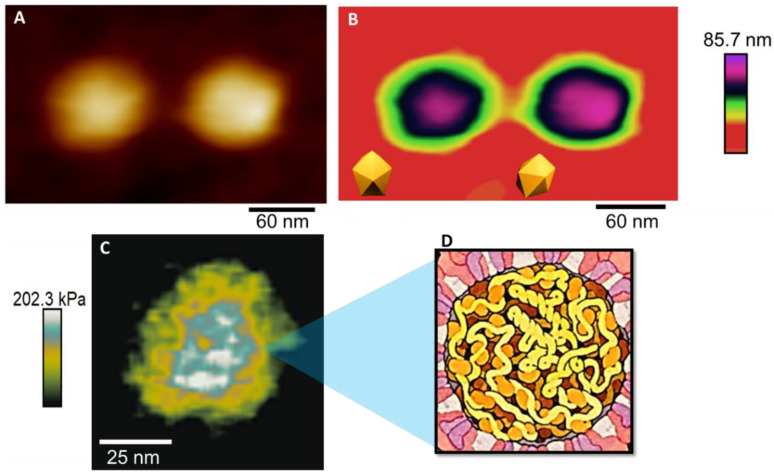
(**A**) Heightmap of two ZIKV particles; (**B**) The same particles with a different height scale contrast, evidencing the icosahedral type of the adsorption pattern. (**C**) Young Modulus map showing internal structure arrangement. (**D**) Pictographic representation of the internal assembly of the ZIKV [35].

The results in Figure 2A,B show the shape of the virus adsorbed on a glass substrate. It is possible to observe a spherical shape, which is very common for the virus [33]. In Figure 2B, it is possible to observe icosahedral faces, as followed by Zandi et al. [34].

**Figure 3:**
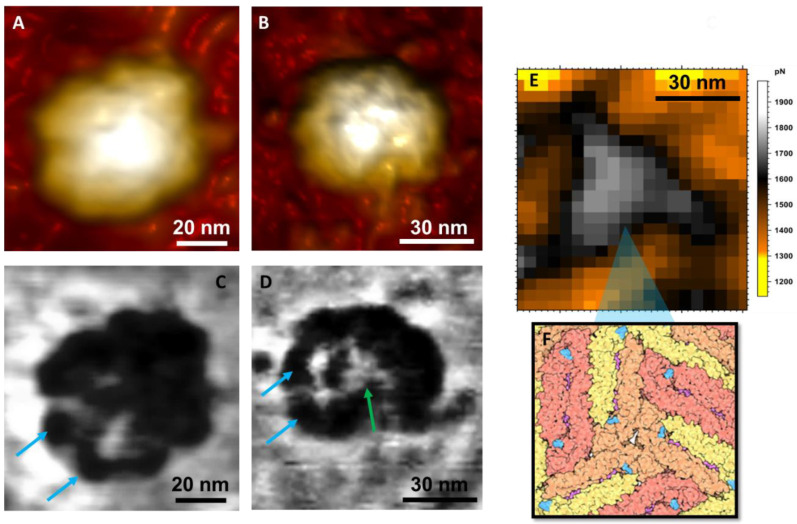
(**A**,**B**) Topographic maps of AFM revealing the viral ultrastructure and particle shape of ZIKV; (**C**,**D**) their respective adhesion maps. (**E**) Closeup adhesion map, showing the correlation between the triangular-shaped structure observed and the model based on cryo-EM results in (**F**) [42]. The blue arrows on both (**C**,**D**) maps indicate the typical bumps of the mature ZIKV particle. The green arrow (**D**) points to the triangular conformation of the closely fitted E protein structures.

Not much has been studied about the mechanics of ZIKV capsids, but physical virology studies suggest that, in general, viral capsids are expected to be more rigid than the membrane because it is the last phase of protection of the viral genome, especially after maturation [36]. In addition, for the flaviviruses, the capsid is reported to be one of the few viral proteins that have been shown to leave the replication compartments and enter the cell nucleus during infection [37], so it should demonstrate greater endurance than the other structures of the viral particle. This is what the Young Modulus (YM) map confirms in Figure 3C. After being stressed multiple times, the outer layer of this virus collapsed, exposing the internal configuration of the ZIKV particle. The Young Modulus is the proportionality constant of a material’s stress-strain relationship, and its maps give information about the elastic nature of the material. Different materials have different contrasts on the map, with stiffer materials having higher YM values and softer materials having lower YM values [38]. In the map of Figure 3C, it is possible to observe the different contrasts in the viral particle, which shows the composition of different materials. In addition, we can state that the internal structure is stiffer than the blue and white contrasts, which on the scale are the colors for higher values of YM in kPa. Opposingly, we have a green–yellow contrast for the outer structures, representing the Young Modulus’s lower values, defining a softer structure for the outside structure.

The adhesion analysis maps revealed more information about the protein arrangement on the virus surface. Figure 3A,B show topographic maps are evidencing the ‘bumpy’ characteristic of the protein arrangement on the virus surface [6]. The envelope protein E is responsible for this viral grouping, tending to form triangular structures, which act as a “shield” against antibodies [4]. In Figure 3C,D, distinct contrasts on the surface demonstrate the fusion of viral proteins that, after the maturation process, are grouped and organized in triangular structures on the virus’s surface (Figure 3E,F). Differences in the adhesion maps’ contrasts may be related to surface charge distribution [22]. For the ZIKV, these distributions could have two possible factors: (i) due to changes in the proteins during the inactivation process, and (ii) they can be related to protein conformations, i.e., monomeric or dimeric conformations [39,40,41].

**Figure 4:**
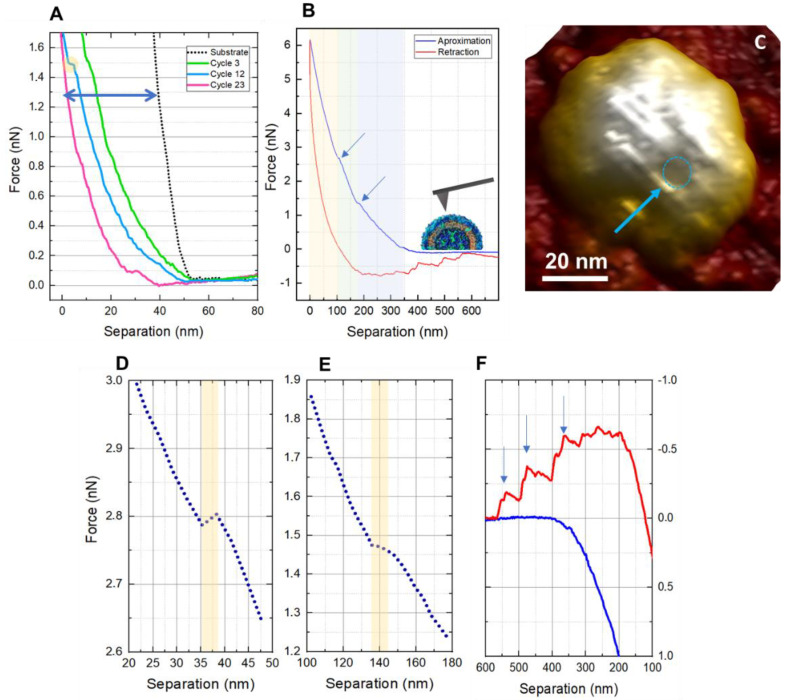
Zika viral particle indentation curves. (**A**) reference curve obtained during the cycles of approach and retraction, demonstrating that as the cycles increase, the curve also tends to lengthen. (**B**) Graph of the Force vs. Separation effected by a single stimulus (cycle) shows that the blue trace represents the approach curve, while the red indicates the retraction curve. The yellow, green, and blue tracks relate to the viral particle’s three different types of materials. The blue arrows in detail of the contact ramp on the approach curve highlight the rupture plateau. (**C**) The image in the inset represents the ruptured region of the structure of the virus membrane, where the blue circle suggests a hole formed by the proteins of the outer layer. (**D**,**E**), related to the shell breakage, represented by the yellow band. (**F**) Tethering events (blue arrows) are observed in the retraction curve (red), matching that ZIKV has only its outermost layer of protein.

Indentation measurements are demonstrated in Figure 4. It is possible to observe that ZIKV particles are more resistant to mechanical stress when compared to other viruses such as SARS-CoV-2 [22]. In Figure 4A, the indentation curves represent the behavior of the viral particles after continuously applied load cycles compared to the reference force curve performed on a glass (non-indentable) substrate (black dotted line). The step (yellow circle) on the curve is related to the 12th cycle, with force values of about 1.5 nN, associated with the rupture of a structural layer [43]. In Figure 4B, it is possible to observe from the approximation curve (blue) that the trigger force to scan the ZIKV can go up to 6 nN when a second plateau is observed at ~2.8 nN. The blue section comprises 5% to 25% of the approach curve and has a measured Young Modulus of about 234 kPa. The green section, in the middle, refers to 25% to 45% of the curve and has a measured YM of 534 kPa. For the yellow section, representing 65% to 85% of the curve, the measured YM was 894 kPa. The curve was linearized by the DMT model [44].

Scans after the fatigue tests showed deformations on the viral surface, confirming that the ZIKV does not have the self-assembly ability (Figure 4C). The plateaus are isolated in Figure 4D,E. The thickness of the ruptured layers is 5.02, and 10.71 nm for the force plateaus shown in Figure 4D,E, respectively. In Figure 4F, we have tethering events on the retraction curve (red) that can be observed in this virus precisely because its outer structure is entirely protein. When the tip is retracted, some portions of protein couple on the AFM probe, and the teeth-like events on the retraction curve represent the pulling forces between what is attached to the probe and what remains on the sample [45,46].

Finally, this result demonstrates that investigation of protein interactions and different viral symmetries (in the case of ZIKV, an icosahedral symmetry) can display a complex range of pathways for viral behavior, leading to a variety of nano-bio-oriented applications [47,48]. Structural proteins can also be targeted by antiviral agents because, in most cases, they are singular to the pathogen and, in general, have no similarities with another human target [49].

## 4. Conclusions

In this study, we used AFM to evaluate the ultrastructure and nanomechanical properties of ZIKV viral particles. The high-resolution topographic maps revealed a viral particle with a mean size of 54.53 ± 6.9 nm. Moreover, it demonstrated that a force of 1.5 nN is necessary to disrupt a first layer and 2.8 nN for a second layer, corroborating the high mechanical resistance. In addition, the thickness of these layers was measured. The protein that protects the ZIKV is molded according to the stimuli, which configures a promising scenario regarding the sequencing of a vaccine. By weakening the structure of ZIKV, it does not present a self-recovery in a short time experiment (~1 min), as already seen in the literature for other types of the virus. The adhesion maps revealed details of the viral ultrastructure that were not seen in the height maps. Variations in elastic modulus are due to the size of the viral capsid—the smaller, the harder—and its external thickness, which again depends on how the protein arrangement is constituted. Young’s module provides a very detailed comparison as it is an intrinsic element of the material, which, as we noted, can be different depending on how the protein “E” remodels. This constituted a challenging analysis since there is a change between intra-capsid interactions—where there is a change of binding forces, for example, by Van der Waals—which explains the increase in capsid rigidity during different indentations.

## Data Availability

The datasets generated during and/or analysed during the current study are available from the corresponding author on reasonable request.
